# Academic Primer Series: Five Key Papers for Consulting Clinician Educators

**DOI:** 10.5811/westjem.2016.11.32613

**Published:** 2017-01-20

**Authors:** Teresa M. Chan, Michael Gottlieb, Antonia Quinn, Kory London, Lauren W. Conlon, Felix Ankel

**Affiliations:** * McMaster University, Department of Medicine, Division of Emergency Medicine, Hamilton, Ontario, Canada; † Rush University Medical Center, Department of Emergency Medicine, Chicago, Illinois; ‡ SUNY Downstate/Kings County Hospital Center, Department of Emergency Medicine, Brooklyn, New York; § Thomas Jefferson University Hospital, Department of Emergency Medicine, Department of Data and Informatics, Philadelphia, Pennsylvania; ¶ University of Pennsylvania, Department of Emergency Medicine, Philadelphia, Pennsylvania; || HealthPartners Institute, Health Professions Education, Bloomington, Minnesota; # University of Minnesota Medical School, Department of Emergency Medicine, Minneapolis, Minnesota

## Abstract

**Introduction:**

Clinician educators are often asked to perform consultations for colleagues. Invitations to consult and advise others on local problems can help foster great collaborations between centers, and allows for an exchange of ideas between programs. In this article, the authors identify and summarize several key papers to assist emerging clinician educators with the consultation process.

**Methods:**

A consensus-building process was used to generate a list of key papers that describe the importance and significance of educational consulting, informed by social media sources. A three-round voting methodology, akin to a Delphi study, determined the most impactful papers from the larger list.

**Results:**

Summaries of the five most highly rated papers on education consultation are presented in this paper. These papers were determined by a mixed group of junior and senior faculty members, who have summarized these papers with respect to their relevance for their peer groups.

**Conclusion:**

Five key papers on the educational consultation process are presented in this paper. These papers offer background and perspective to help junior faculty gain a grasp of consultation processes.

## INTRODUCTION

Solving educational problems through educational consulting is recognized as a key skill for clinician educators.^1^ Along with other skills such as teaching,^2^,^3^ research and scholarship,^1^,^3^–^5^ faculty development,^1^,^2^ and leadership,^2^,^6^ performing consultations for educational problems is an expected skill of a clinician educator.

The clinician educator’s role in a consultation is to integrate education theory with practice.^2^,^5^ The ability to apply education theory to practice is a key skill that differentiates a clinician educator from a clinician teacher (i.e., a clinician with only supervision or teaching roles).

Previously, we have discussed the role of education scholarship in the careers of clinician educators.^7^ While engagement in scholarship is important, it is equally important for clinician educators to assist in translating the work of other education researchers and scholars into everyday practice.^5^ Functioning as a problem-solver or consultant is one way in which one can participate in this act of knowledge translation.

The *Academic Life in Emergency Medicine* (ALiEM) Faculty Incubator was created to train early career educators in developing the theoretical background needed to effectively complete educational consults. During our one-year experience, we created a one-month module focused on the art of performing the education consult. This paper is a synthetic, narrative review that highlights some important literature that may assist junior educators as they begin acting as consultants for local and external groups.

## METHODS

In the fourth month of the 2016–2017 ALiEM Faculty Incubator (June 2016), the topic of education consultation was discussed. We monitored the proceedings of this group of educators from July 1–31, 2016. The discussion was allowed to unfold asynchronously; during this process, we gathered the titles of papers that were cited, shared, suggested, and discussed within the online discussion forum. Multiple participants in the Faculty Incubator (both junior and senior members) contributed papers to the discussion. This list was then augmented with a call for suggestions on Twitter. We “tweeted” requests to have participants of the #FOAMed and #MedEd online communities provide suggestions for important papers on the topic of education consultation and the role of the clinician educator.

The list of papers was compiled for the authors, who subsequently conducted a three-round voting process, inspired by the Delphi methodology. This was not a traditional Delphi methodology since our selection panel comprised of both novices (i.e. junior faculty members, participants in the Faculty Incubator) and experts in the field (i.e. experienced clinician educators, all of whom have published >10 peer reviewed publications, who serve as mentors and facilitators of the ALiEM Faculty Incubator). We intentionally sought to involve both junior and experienced clinician educators to ensure we selected papers that would be of use to a spectrum of educators at different career stages. The three phases for this multi-round consensus building process consisted of the following:

1. Round 1: A first round where each paper was voted along a seven-point scale, with a “1” being Unimportant for Junior Faculty (Unlikely to Significantly Impact Junior Faculty) and a “7” being Essential for Junior Faculty (Illuminating, Highly Useful).
2. Based on the authorship group’s first-round scores, the participants were subsequently asked to vote on the papers that they thought should be included it the top five papers, but were allowed to endorse more than five papers in total.
3. In the third round, the same group was asked to review the percent endorsement for each paper and vote on ONLY five papers that should be recommended in the final paper.

After reviewing the papers in full, there were two papers excluded from this commentary, since the type of consultation discussed in those papers was not within the scope of this paper.

## RESULTS

Our initial review of the ALiEM Faculty Incubator discussion thread yielded a total of 18 articles, which were mentioned by mentors and the junior faculty incubator participants. The social media call added one additional paper. The three-round voting procedure allowed our team to generate a rank-order listing of all papers in order of relevance, from the most important to the least important. Three papers were excluded as irrelevant after consultation with the entire authorship group, as they pertained to clinical consultations rather than educational consultations. The citations and our ratings of these papers are listed in the Table.

## DISCUSSION

The following are a summary of the top papers accompanied with commentaries on their relevance to both junior faculty members, as well as potential considerations for faculty developers when discussing these works.

### 1. Sherbino J, Frank JR, Snell L. Defining the key roles and competencies of the clinician-educator of the 21st century: a national mixed-methods study. *Acad Med*. 2014 May;89(5):783–9.^5^

#### Summary

The current medical education environment requires increased accountability and revision of accreditation standards. As a result, formal medical education relies on a key group of clinician educators or medical consultants to serve as leaders in medical education. This study attempts to provide a formal definition for medical education consultants and describes the core competencies of a clinician educator. Clinician educators must be active in their practice, apply “education theory to teaching and learning,” and engage in educational scholarship. Scholarship is not limited to formal research, but includes the scholarship of integration, application, and teaching.^21^ In order to achieve these traits, medical consultants require additional training in medical education, such as advanced degree programs or continuous faculty development. Furthermore, clinician educators possess excellent communication skills and participate in curriculum development and assessment, with a firm basis in established educational theory. 5

#### Relevance to Junior Faculty Members

Junior faculty may not have the confidence to provide medical education advice to their colleagues. However, this article confirms that the majority of medical education leadership felt that junior faculty are qualified to be clinician educators. Clinician educators are in high demand, with 40% of respondents indicating training programs will require education consultation at least one half-day per week.^5^ To be a respected consultant, the junior faculty member must receive some form of advanced medical education training, such as certificate programs or organized faculty development.^5^ A firm understanding of education theory differentiates clinical educators from clinician teachers.

#### Considerations for Faculty Developers

Often a junior clinician educator may not initially imagine his- or herself as a person whom others might turn to for assistance or advice on medical education matters. This paper explains how education consultation fits into the job of a clinician educator.^5^ Providing junior colleagues with this information early in a faculty development program may help them consider what skills and expertise they must gain so they can be prepared to be a better consultant later in their career.

### 2. Brown T. Design thinking. *Harv Bus Rev*. 2008 Jun 1;86(6):84–92.^8^

#### Summary

This paper is a modern classic from the *Harvard Business Review*. Tim Brown explains the basic concepts behind a major business consulting approach that has arisen in the past 20 years: Design thinking.^8^ Design thinking is a human-centered business model that emphasizes the need for input from a wide range of users, fluency in ideas, and early, rapid prototyping so as to isolate the best solution to a problem. This paper also provides numerous examples of the use of design thinking both within and outside of the business world, describing its application in the design of a new approach to patient care transitions within a large healthcare system, the development of a new surgical tool, and expanding eye care to locations with poor healthcare access.^8^

#### Relevance to Junior Faculty Members

This article may be particularly valuable to junior faculty members as they are looking to improve upon existing or create new curricular models. Before initiating a new curricular change, it is important to perform a *Needs Assessment*.^22^ Design thinking emphasizes the importance of involving a variety of end-users in the needs assessment and development stages to identify potential challenges and solutions. For example, when designing a new approach to patient care transitions, it would be valuable to involve residents and attending physicians from that department, nursing staff, consultants, and even patients to best understand the various components and challenges involved. It is also important to seek out *extreme users* (i.e. users who are at opposite extremes) and learn the different problems and workarounds they have developed. Finally, design thinking is a fluid and continuous process. While the process is often described in a series of stages, one must be cognizant that this should be a continuous and inter-linked process that thrives on broad ranges of ideas with frequent and rapid prototyping.

#### Considerations for Faculty Developers

Senior faculty members looking to use this paper for faculty development may find it useful to practice brainstorming and learner-centered interviewing with junior clinician educators via simulated exercises. Developing and affirming creativity is key for increasing fluency of ideas, and many junior faculty members may be initially uncomfortable or unfamiliar with these techniques. For more education-specific design thinking readings, senior faculty members may want to review resources from the webpage *Design Thinking for Educators* (http://www.designthinkingforeducators.com/), which contains free videos, examples, and a downloadable educator’s toolkit in PDF format. Mr. Brown’s paper is most useful as a starting point to open up a discussion around user-centered design, which has applications in medical education (i.e. learner-centered design) and health care (i.e. patient- or family-centered design).

### 3. Madsbjerg C, Rasmussen MB. An anthropologist walks into a bar. *Harv Bus Rev.* 2014 Mar 1;92:80–8.^10^

#### Summary

Finding a way to sift through complex social challenges is the primary focus of this article. The authors provide examples from the business world (e.g. a brewery, a toy maker, and a medical supply company) to show that everyday assumptions about human motivation can frequently be misguided if not directly anathema to the underlying truth. To solve these issues, one requires a systematic approach relying on empathy and the refusal to be guided by a priori thinking, known as *sense-making*. The first and most important step is to reframe the problem. Whereas most problems are seen as dichotomous issues of fact or fiction, sense-making requires a recalibration, looking at the subjective experience of the end-user. By attempting to ascertain the underlying motivations of the intended audience, one is better able to see the holes that prevented current practice from fulfilling expectations. After the underlying question has been found, one needs to gather diverse qualitative data about the issue, look for themes that emerge, and build upon that foundation.

#### Relevance to Junior Faculty Members

Frequently, novice educators are left without a framework of how to approach problems to which they are tasked with solving. Whereas their preclinical, clinical, and graduate medical education may have prepared them to decipher complex biostatistical methodologies and critically appraise the merits of quantitative research, what is lost is the ability to troubleshoot social issues. To the novice educator, learning about empathy is rarely, if ever, broached in relation to patient interactions. Educators must be able to find holes in curriculum that they may not be aware or have experienced. Only through the eyes of their learners, can one gain the insight to make lasting and impactful changes. For example, a lecture-based curriculum for ultrasound may seem relevant to a teacher since she can connect the images to their reasoning, but if the learners desire training on image acquisition, the hands off style may be underappreciated and wasteful.

#### Considerations for Faculty Developers

This paper expands upon the concepts described in the earlier paper by Tim Brown.^8^ One of the key elements of great human- (or learner-) centered design is the ability to empathize and understand the needs of those for whom you are designing. Faculty developers who are seeking to teach junior faculty members about design thinking processes can use this paper to introduce some useful data collection techniques that assist in the evaluation of end-user needs. Those senior faculty familiar with qualitative methods will note that many of the techniques mentioned in this paper are consistent with those from social sciences, such as anthropology or sociology. This paper may serve as a good launching point for discussing what is truly needed in the local needs assessment phase of the curriculum design (as described by Kern^22^), or how one might diversify his or her techniques when gathering user-centered data during a robust program evaluation procedure.

### 4. Turner AN. Consulting is more than giving advice. *Harv Bus Rev*. 1982 Sep–Oct;60(5):120–9.^9^

#### Summary

Despite having been published 30 years ago, this classic business article still rings true for consultants. The article discusses the hierarchal pyramid for consulting, beginning with providing simple solutions and progressing through solving more complex problems, assisting with implementation, and eventually helping clients to self-diagnose problems and improve their own efficiency.^9^ Some valuable points presented throughout the article include the importance of ensuring that the question is appropriate for the problem; understanding institutional limitations to ensure that solutions are feasible; and involving multiple levels of stakeholders to increase insights and buy-in.

#### Relevance to Junior Faculty Members

While this article was initially written for the business consultant, one could readily see the application to the education consultant, as well. Mirroring Bloom’s Taxonomy,^23^ this paper emphasizes the progressive levels of knowledge acquisition and self-direction that the consultant or educator wants the learner to achieve. As an educational consultant, it is important to remember that the goal is not merely to answer the question, but to assist the “consultee” in finding the answers and expanding their own knowledge and skill sets.

#### Considerations for Faculty Developers

This article offers an important hierarchical model of consultation sophistication that serves as a useful framework for faculty developers to guide junior educators. Faculty developers can use this framework to match the development plan and readiness of programs to engage in consultations of value. It also describes the importance of matching the readiness of the programs asking for consultations with the preferred method of consultation for the consultant. Ultimately, the article provides a stepwise approach to consultants wishing to turn programs into full-fledged learning organizations and permanently improving organizational effectiveness.

### 5. Levinson W, Rubenstein A. Integrating clinician-educators into academic medical centers: challenges and potential solutions. *Acad Med.* 2000 Sep;75(9):906–12.^4^

#### Summary

This commentary highlights challenges of integrating clinician educators into the standard promotional track at academic medical centers. The authors cite that an increasing proportion of faculty at academic medical centers (AMCs) are primarily spending their time working clinically, which is suggested to be a direct result of a changing economic structure for AMCs. These clinical educators are the foundation for education at AMCs. While colleagues, residents, and students appreciate their work, institutional credit is less common. Barriers to institutional recognition include the requirement for regional and national reputations among clinician educators, the lack of valid measurements for teaching activities, and the lack of training opportunities for junior faculty members. Potential solutions identified by the authors include hiring clinician educators as short-term employees with the intention of hiring new faculty every few years, as well as committing to develop a core group of clinician-educators that will focus on institution-specific educational programs. Analogous to this, the development of the education researcher will augment the growth of the core group of clinician educators.

#### Relevance to Junior Faculty Members

The majority of junior faculty members at AMCs will be clinician educators. It is important to understand requirements for promotion, as clinician educators often have difficulty advancing within this track. The authors identify the possibility of hiring a new group of clinical educators every few years to address the difficulty in promotion as well as developing a core set of clinician educator researchers. Junior clinician educators should be aware of this as they develop novel educational programs and seek to publish the work they are doing. Longevity as a clinician educator will likely come to those who commit to developing scholarly skills within medical education, but also by mentoring new junior colleagues once established.

#### Considerations for Faculty Developers

This article presents a comprehensive review of the challenges for promotion for clinician educators and focuses on three themes: (1) regional and national reputation; (2) lack of metrics to measure educational impact;,and (3) challenges in researching educational innovations.^4^ It gives examples of two institutions that have addressed these challenges. This articles can help faculty developers can focus on development plans for each of these areas when mentoring junior faculty and help inform junior faculty in the clinician educator track of the historical context of common for promotion challenges for clinician educators.

#### Excluded papers

During this month, our Faculty Incubator discussed the topic of consultations within the clinical context as well. During our online discussion, we discussed two papers^13^,^11^ that discuss the nature and best practices for consulting colleagues in EM clinical cases. Although these papers are not relevant to our present discussion, the papers were rated initially quite favourably (>4/7 in terms of our initial Likert scale of relevance to junior faculty), and as such we have listed them in the Table.

## LIMITATIONS

Of note this month, the faculty incubator participants and mentors had a wide-ranging discussion that included some papers that may seem irrelevant to readers expecting a paper on educational consultations. That being said, we have elected to be inclusive of all the papers we discussed this month, since some of these papers may be of use to those interested in other more peripherally related topics (i.e. emergency department referral and consultation processes).^13^,^11^

This was not an exhaustive, systematic search of the literature. We attempted to find relevant readings for the Faculty Incubator by performing a search online via Google Scholar looking for any key papers on completing educational consultations. We also attempted to seek assistance with finding more papers by using an open social media call via Twitter using hashtags #MedEd & #FOAMed, but only one additional paper was found. Since the purpose of this paper was to aggregate an introductory set of papers to assist junior faculty members in thinking about the consultation process, we feel that our method allowed us to aggregate papers that would accomplish this feat. Finally, we note that there may be an inherent selection bias of these topics by our junior faculty members who are involved in the Faculty Incubator. Of note, one of the in-person activities for the ALiEM Faculty Incubator 2016–2017 program included a design thinking introduction, which may have affected the selection of papers related to this topic for this paper.

## CONCLUSION

The authors provided a reading list that may be beneficial as an introduction for junior faculty members to better acquaint themselves with consulting on medical education problems. We hope this paper provides junior clinician educators a broad overview of this important topic and makes it more approachable and less intimidating.

## Figures and Tables

**Table:** The complete list of educational scholarship literature collected by the authorship team.

Citation	ROUND 1 initial mean scores (SD) max score 7	ROUND 2 % of raters that endorsed this paper	ROUND 3 % of raters that endorsed paper in last round	Top 5 papers
Brown T. Design thinking. *Harv Bus Rev*. 2008 Jun 1;86(6):84.8	6.2 (1.6)	83.3%	100%	1
Sherbino J, Frank JR, Snell L. Defining the key roles and competencies of the clinician-educator of the 21st century: a national mixed-methods study. *Acad Med.* 2014 May;89(5):783–9.^5^	5.2 (2.0)	66.7%	100%	2
Turner AN. Consulting is More Than Giving Advice. *Harv Bus Rev*. 1982 Sep–Oct;60(5):120–9.^9^	4.8 (1.3)	83.3%	83.3%	3
Madsbjerg C, Rasmussen MB. An Anthropologist Walks into a Bar. *Harv Bus Rev*. 2014 Mar 1;92:80–8.^10^	5.3 (1.9)	83.3%	83.3%	4
Levinson W, Rubenstein A. Integrating clinician-educators into Academic Medical Centers: challenges and potential solutions. *Acad Med*. 2000 Sep;75(9):906–12.^4^	5.2 (1.2)	66.7%	66.7%	5
Kessler CS, Chan T, Loeb JM, et al. I’m clear, you’re clear, we’re all clear: improving consultation communication skills in undergraduate medical education. *Acad Med*. 2013 Jun;88(6):753–8.^11^	5.0 (1.9)	Excluded due to lack of relevance to present review.		
Roberts DH, Schwartzstein RM, Weinberger SE. Career development for the clinician-educator. Optimizing impact and maximizing success. *Ann Am Thorac Soc.* 2014 Feb;11(2):254–9.^3^	5.0 (1.1)	16.7%	0%	
Norman GR. Problem-solving skills, solving problems and problem-based learning. *Med Educ.* 1988 Jul;22(4):279–86.^12^	4.5 (1.8)	33.3%	0%	
Chan T, Orlich D, Kulasegaram K, et al. Understanding communication between emergency and consulting physicians: a qualitative study that describes and defines the essential elements of the emergency department consultation-referral process for the junior learner. *CJEM*. 2013 Jan;15(1):42–51.^13^	4.3 (2.1)	Excluded due to lack of relevance to present review.		
Branch, W. T., Kroenke, K., & Levinson, W. (1997). The Clinician-Educator—Present and Future Roles. *J Gen Intern Med*, 12(Suppl 2), S1–S4.^2^	4.3 (1.0)	50%	0%	
Sherbino J, Snell L, Dath D, et al. A national clinician-educator program: a model of an effective community of practice. *Med Educ Online*. 2010 Dec 6;15.^14^	4.2 (1.8)	16.7%	16.7%	
Steinert Y, Mann K, Centeno A, et al. A systematic review of faculty development initiatives designed to improve teaching effectiveness in medical education: BEME Guide No. 8. *Med Teach*. 2006 Sep;28(6):497–526.^15^	4.2 (1.6)	0%	0%	
Osterberg L, Swigris R, Weil A, et al. The highly influential teacher: recognising our unsung heroes. *Med Educ*. 2015 Nov;49(11):1117–23.^16^	4.2 (1.2)	16.7%	0%	
Leslie K, Baker L, Egan-Lee E, et al. Advancing faculty development in medical education: a systematic review. *Acad Med*. 2013 Jul;88(7):1038–45.^17^	3.3 (1.8)	0%	0%	
Mezrich R, Nagy PG. The academic RVU: a system for measuring academic productivity. *J Am Coll Radiol*. 2007 Jul;4(7):471–8.^18^	3.2 (1.9)	0%	0%	
House J, Santen SA, Carney M, et al. Implementation of an education value unit (EVU) system to recognize faculty contributions. *West J Emerg Med*. 2015 Nov;16(6):952–6.^19^	3.0 (1.4)	0%	0%	
Regan L, Jung J, Kelen GD. Educational value units: a mission-based approach to assigning and monitoring faculty teaching activities in an academic medical department. *Acad Med*. 2016 Feb 2.^20^	2.8 (1.5)	16.7%	0%	
Straus SE, Soobiah C, Levinson W. The impact of leadership training programs on physicians in academic medical centers: a systematic review. *Acad Med.* 2013 May;88(5):710–23.^6^	2.8 (1.3)	0%	0%	
